# New doctor-patient communication learning software to help interns succeed in communication skills

**DOI:** 10.1186/s12909-019-1917-z

**Published:** 2020-01-08

**Authors:** Chao Sun, Junkai Zou, Lanbo Zhao, Qing Wang, Shaozhi Zhang, Qurat Ulain, Qing Song, Qiling Li

**Affiliations:** 1 First Affiliated Hospital, Xi’an Jiaotong University, 277 Yanta West Road, Xi’an, Shaanxi China; 2 Affiliated Tumor Hospital, Xinjiang Medical University, 789 Suzhou East Street, Urumqi, Xinjiang, China; 3 Department of Electronic Engineering, Xidian University, 2 Taibai South Road, Xi’an, Shaanxi China; 4 Cardiovascular Research Institute, Morehouse School of Medicine, 720 Westview Drive, Atlanta, Gorge USA

**Keywords:** Doctor-patient, Communication, Learning software, Communication ability, Interns

## Abstract

**Background:**

Nowadays, the research of doctor-patient communication is becoming increasingly important not only in China but also around the world.

**Methods:**

The study designs a type of learning software to train the interns to advance their communication skills, and whose validity for improving doctor-patient communication in self-controlled trials is evaluated. With the aid of the new learning software, the self-controlled tests were carried out among 183 interns to assess the quality of their communication skill acquisition. The learning effectiveness of the preparation stage, information collection, information given, patient understanding, and inquisition ending was evaluated with the Set Elicit Give Understand End (SEGUE) framework after 3 months of training.

**Results:**

More interns (37.16% vs. 10.98%, *P* < 0.001) could accurately identify the psychosocial or emotional factors contributing to the diseases. An increased number of interns (42.62% vs. 10.40%, *P* < 0.001) were able to openly discuss lifestyle issues and prevention strategies with patients. The study also revealed that interns who had completed training tended to allow patients more time to describe their feelings and concerns about their illnesses. In addition, more of the trained interns roved capable of being caring and respectful to patients and showing empathetic communication behavior (53.01% vs. 26.59%, *P* < 0.001).

**Conclusions:**

The doctor-patient communication software may help the interns known more about communication skills.

## Background

The doctor-patient relationship has become a complex social issue in China due to a lack of trust and mutual understanding between doctors and patients. As the Chinese Medical Doctors’ Association reported, the number of violent attacks on doctors rose from 57 cases to 130 cases between 2010 and 2013 [1]. More than two-thirds of doctors surveyed admitted that tension and conflict exist between doctors and patients.

One study has indicated that 98.47% of hospitals were troubled by medical disputes, 70% of which were caused by ineffective communication between doctors and patients [2]. The doctor-patient relationship in China can be described as patriarchal and is characterized by a doctor-centered mode of communication [3]. Doctors are expected to be the experts who give patients appropriate medical instruction as to what to do. Patient-centered interviewing is unusual in China. Patient-centered communication between doctors and patients is essential for the delivery of high-quality patient care [4]. Unlike other countries, a dilemma is often faced by the Chinese doctors [5]. Most Chinese families ask physicians not to reveal the diagnosis and prognosis to patients. However, more and more patients want to be fully informed, and the right of being informed has been regulated by law. Competent communication by doctors can improve health outcomes, enhance patient satisfaction, and contribute to doctors’ job satisfaction [6]. Conversely, poor communication and attitude between doctor and patient may constitute the most frequent underlying cause of malpractice litigation, complaints against doctors, and non-adherence to medication regimens [7, 8]. Therefore, the development of effective communication skills is an important part of becoming a good doctor. There is strong evidence that appropriate teaching can help medical students acquire and retain these skills [9–12].

Thus, the development of strategies targeting at strengthening communication has become a central topic in clinical and social research. About one decade ago, the theories and methodologies of doctor-patient communication skills training (CST) had been introduced in China. For instance, the Calgary-Cambridge Guide [13] and the SEGUE Framework [14] had been modified to conform to the Chinese culture. In 2003, the educators of Nanjing Medical University developed and deployed the first doctor-patient communication curriculum. Nonetheless, recent research [2] shows that only 40% of Chinese medical schools included doctor-patient communication in their curriculum, and most of the CST was an optional course. Furthermore, it is hard for residents and practicing physicians to receive CST continuing education. Compared to the identified substantial need for doctor-patient CST, the resources and endeavor devoted to it remain inadequate. As was suggested in Tu et’s [15] investigation on outpatient communication in a large hospital in China, there is an extreme inequality of power between Chinese doctors and patients at the individual level. More public education and professional skills training are needed to improve the communication and promote mutual understanding between patients and doctors.

In addition to CST courses, simulated patients (SPs) are an important didactic tool in the education of medical students [16]. Medical curricula throughout the world consist of such simulation trainings [17–19]. In spite of this, Bokken L [20] pointed that there appear to be no clear standards with regard to effective feedback training for SPs. Moreover, there is no solid scientific basis in terms of the processes by which feedback is provided by SPs and the selection of domain(s) in which SPs give feedback often.

Recently, some researchers [21, 22] have reported that 3D printed models could improve the patients’ understanding and compliance in surgery. However, some problems have also been identified, for example, this method is only suitable for some communication scenarios, and it consumes financial and material resources, and does not have the feasibility of wide application.

Therefore, in order to popularize the doctor-patient communication education and make learning methods more diversified, we try to combine online learning with communication skills learning. Under the circumstance, a new doctor-patient communication learning software is designed and produced in the paper. Meanwhile, its effectiveness has also been tested. It is a new method that was by no means available from domestic and overseas literatures [12, 23].

### Methods

#### Design and preparation of new doctor-patient communication learning software

The doctor-patient communication learning software was written in the C++ language, the development process of which was divided into three sequential stages: business modeling requirements phase, design phase and implementation phase of the project. The backend database involved the open source mysql, and the front end was rendered with Hyper Text Markup Language 5 technology. With its background supported by the Thinkphp framework for Model-View-Controller, the site was built on Sina cloud to facilitate program monitoring and subsequent migration. The basic information and answer records of all users were stored in the website database so that further data analysis could be carried out. The doctor-patient communication learning software was designed with the aim of transforming the traditional model of communication skills training for Chinese medical students in hopes that its rich and colorful forms could shed light on doctor-patient communication training program provided by the following link: http://yhgt.applinzi.com/.

**Fig. 1:**
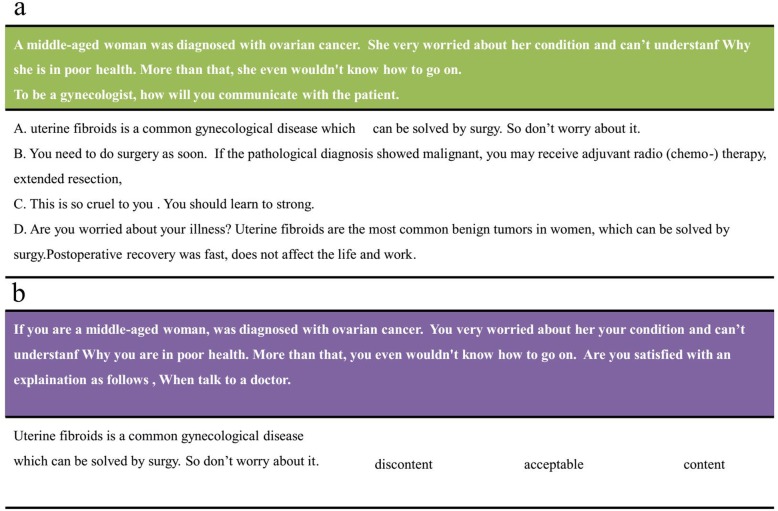
An example of scenario question and related question. If one chooses A in 1a, then the updated question accordingly is what one observes in 1b

The software mainly consists of two modules, namely a random module and a scenario module. The random module teaches students theoretical knowledge of doctor-patient communication skills, while the scenario module allows the learners to apply the knowledge gained in the random module to real case studies. There are some theoretical questions with 4 or 5 choices for students to choose in random module. Each question has one or more correct answers. As far as the scenario module is concerned, however, the questions from clinical cases are more practical, and each choice has a corresponding score, which means there are no absolutely correct answers. Once the students completed the test questions in the scenario module, the updated questionnaires were generated based on the answers (The example is shown in Fig. 1). Meanwhile, related questionnaires were emailed to friends or family who could play the role of the patient.

In clinical practice, SPs technology [24] is extensively applied in teaching and evaluation as SPs are able to provide credible interactions for students. However, without standardized training, friends or family as simulated patients are likely to be more real, effective and closer to the actual situations. In this sense, the evaluation was more objective and sufficient.

All clinic cases in the scenario module were collected at the First Affiliated Hospital of Xi’an Jiaotong University from January 2015 and August 2015. Referencing more than 20 books and research articles about communication and medical ethics, our team members designed the test questions, which were then reviewed and revised by training experts from First Affiliated Hospital of Xi’an Jiaotong University.

#### Participants and groups

The study protocol was approved by the Ethics Committee of the First Affiliated Hospital of Xi’an Jiaotong University in China, and the research was conducted in the Gynecology and Obstetrics Department from 2015 to 2016. The design involved a self-controlled trial with one group. One software developer and six gynecologists (chief physician, associate chief physician, postgraduate [1:1:4]) participated in the research.

A total of 183 interns (79 males and 104 females, 4 ethnic minorities) from the First Affiliated Hospital of Xi’an Jiaotong University were selected as the research subjects whom were randomly divided into two groups. Of these 183 students, 10 missed the base value test and were excluded from analysis.

#### Research protocol

Self-controlled trial: The students’ basic levels of doctor-patient communication were tested through the “doctor-patient communication scale” in May 2015; students who completed the doctor-patient communication skills training through the “doctor-patient communication software” were measured again in December 2015.

The SEGUE framework [14] was chosen as the “doctor-patient communication scale”.

#### Measures and statistical analyses

The data analysis and statistics were performed with Statistical Program for Social Sciences (SPSS) software, version 10.0. A comparison between the pre-test and post-test scores was performed through the independent-sample t test. Statistical significance was accepted at *P* < 0.05.

## Results

### New doctor-patient communication learning software

More than 20 professional books were consulted about doctor-patient communication, medical ethics and other subjects. Based on this, 150 theoretical questions (including regulations, skills, principles, etc.) were formulated according to relevant knowledge, and 100 questions were selected including 85 single choice questions and 15 multiple choice questions. More than 50 clinical cases were collected from internal medical departments (Department of Cardiology, Respiratory Medicine, Gastroenterology), Surgery Departments (Department of Urology, Department of Orthopedics, Department of General Surgery), Department of Obstetrics and Gynecology, Department of Pediatrics and other disciplines; 120 test questions and patient satisfaction questions were created from parts of 30 of the 50 clinical cases.

According to our design idea (as described in materials and methods), it took as long as two months to complete the original doctor-patient communication learning software. Two modules and an evaluation system are available in this software: random pattern, scenario pattern and a patient satisfaction questionnaire.

**Fig. 2:**
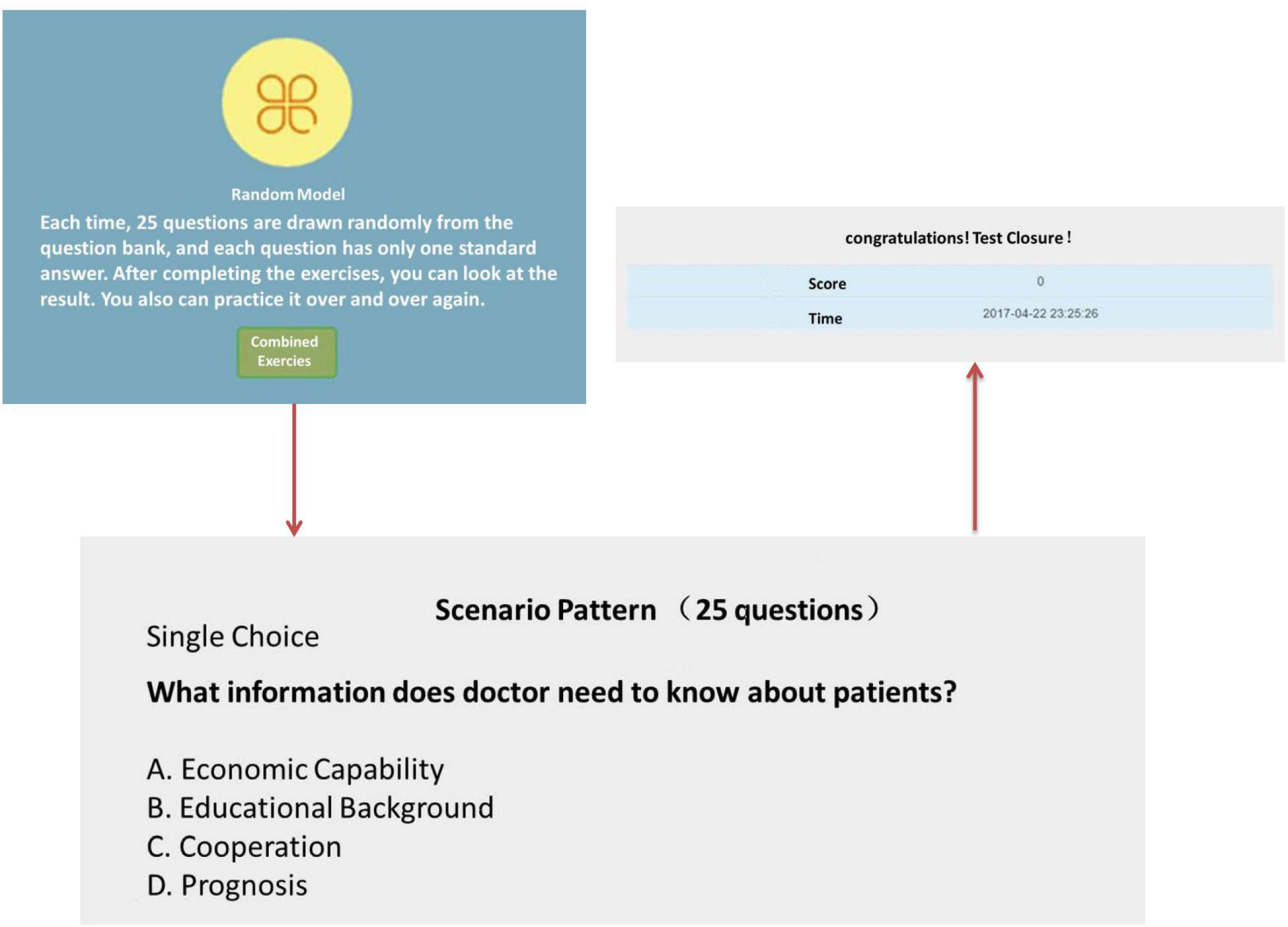
Random pattern. In this pattern, students could learn repeatedly. Each time, 25 questions were prepared randomly in the system, and only a standard answer was set for students

***Random module (***Fig. 2***):*** There were 100 doctor-patient communication learning questions. Each time, 25 questions were randomly prepared in the system and a standard answer was set for students. After completing the exercise, students could view the results and repeatedly practice.

**Fig. 3:**
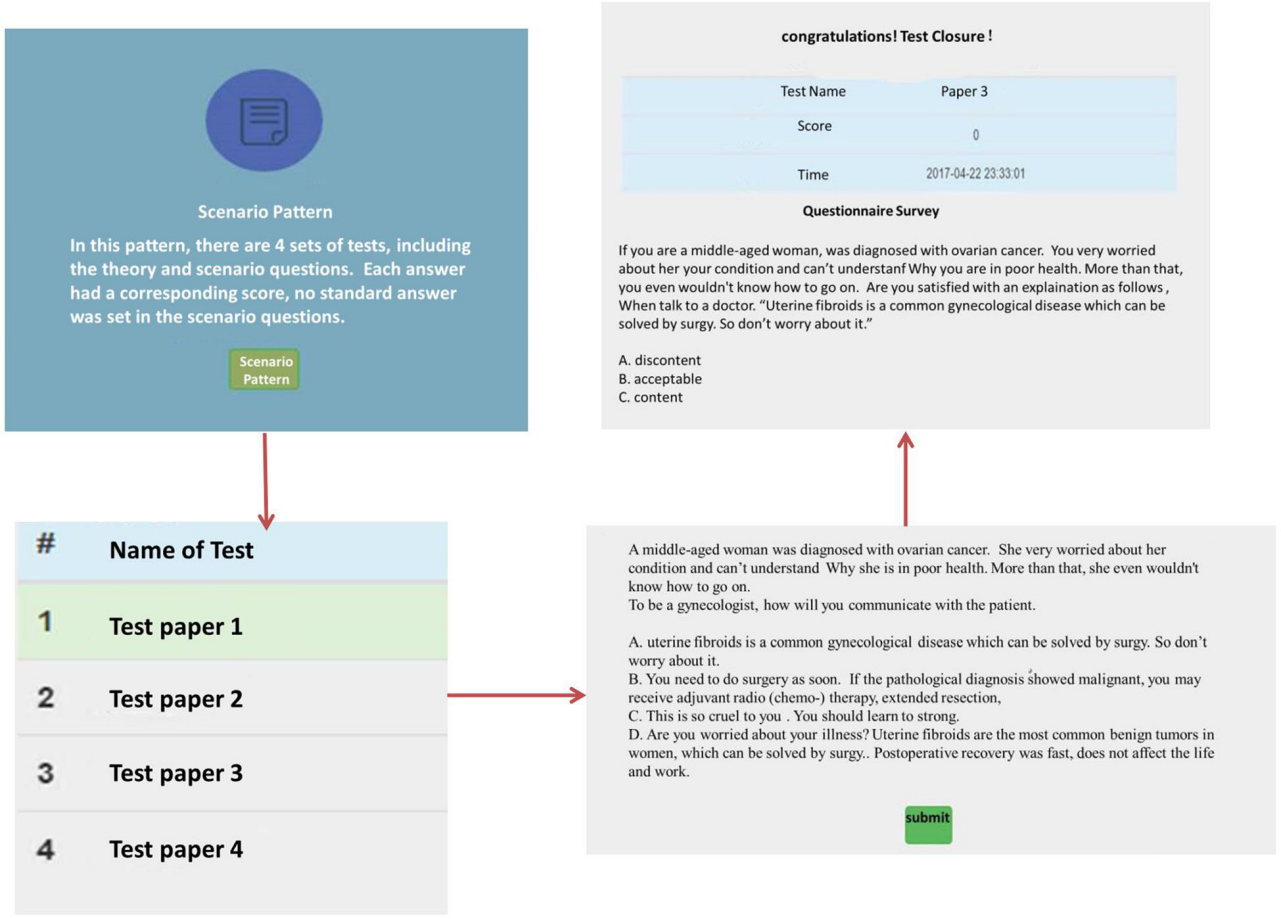
Scenario pattern. In this pattern, students could learn the clinical communication cases. An updated score accordingly was set to every answer, and no standard answer was set in the scenario questions

***Scenario module (***Fig. 3***):*** In this module, there were 4 sets of tests involving the theoretic and scenario questions. An updated score was set accordingly to each answer; no standard answer was set in the scenario questions.

***Patient satisfaction Questionnaire:*** Once the students completed the test questions, the related questionnaires were generated according to the answers. Students could not only invite patients to answer the questionnaire but also send the questionnaire to their relatives and friends by e-mail to answer it from the patient’s point of view. It is an excellent assessment for the scenario module.

### Self-controlled trial (Table 1)

For the first trial, a total of 183 questionnaires were issued, and 173 were actually recovered, at a return rate of 94.5%; 183 questionnaires of second trial were issued, and 183 were actually recovered, with a return rate of 100%. All the 183 interns completed the doctor-patient communication software learning.

**Table 1:** Statistical Reports about Controlled Trail

	Base score	Test score	The value of Χ^2^	The value of *P*
The number of students	173	183		
Preparation Stage				
courtesy title	156 (90.17)	179 (97.81)	9.353	0.002
Inquiring about reasons for interrogation	147 (84.97)	151 (82.51)	0.394	0.530
Introduce operation process	17 (9.83)	23 (12.57)	0.670	0.413
Build trust relationships	30 (17.34)	32 (17.49)	0.001	0.971
Protect patient privacy	58 (33.53)	62 (33.88)	0.005	0.944
Information Collection				
To guide the patients to talk about	20 (11.56)	28 (15.30)	1.066	0.302
Systematic inquiry for Disease	154 (89.02)	158 (86.34)	0.589	0.443
Asking about psychological factors	19 (10.98)	68 (37.16)	32.997	< 0.001
Discuss the diagnosis and treatment	150 (86.71)	147 (80.33)	2.616	0.106
Quality of life	56 (32.37)	57 (31.15)	0.061	0.804
Talking about healthy living	18 (10.40)	78 (42.62)	46.871	< 0.001
Avoid inducing questions	57 (32.95)	34 (18.58)	9.649	0.002
Give patients time to express	53 (30.64)	77 (42.08)	5.021	0.025
Listen carefully and give feedback	28 (16.18)	35 (19.13)	0.528	0.467
Verify patient information	71 (41.04)	89 (48.63)	2.072	0.150
Information Given				
Interpretation basis	26 (15.03)	37 (20.22)	1.644	0.200
Informing physical condition	15 (8.67)	72 (39.34)	45.312	< 0.001
Comfort and encourage patients	11 (6.34)	18 (9.84)	1.437	0.231
Adjust the way of interpretation	55 (31.79)	70 (38.25)	1.629	0.202
Patients Understanding				
Identify patient efforts	57 (32.95)	55 (30.05)	0.345	0.557
Observe the patient’s suggestion	35 (20.23)	53 (28.96)	3.643	0.056
Expressing concern	46 (26.59)	97 (53.01)	25.820	< 0.001
Maintain respectful tone	161 (93.06)	162 (88.52)	2.178	0.140
Inquisition Ending				
Make sure the patient has no problem	49 (28.32)	52 (28.42)	0.069	0.793
Describe the treatment plan	57 (32.95)	55 (30.05)	0.345	0.557

Of the 183 students involved, 10 did not or not fully participate in the course. Thus, data sets of *N* = 173 students (75 males and 98 females, 4 ethnic minorities) were available for analysis. The mean age of participants was 21.5 years (±2.40).

In order to judge the ability and function of the software in improving the communication level, 25 communication skills of SEGUE were tested by t test before and after learning. Seven results became significant. Specifically, they are the Courtesy title, Avoid inducing questions, Give patients time to express, Inquiring about healthy living, Talking about healthy living, Informing physical condition and Expressing concern.

However, through the study of the software, there was no significant improvement in most communication skills (18/25) before and after learning. These skills are listed as follows: Inquiring about reasons for interrogation, Introduce operation process, Build trust relationships, Protect patients’ privacy, Guide patients to talk about their symptoms, Systematic inquiry for Disease, Discuss about diagnosis and treatment, Quality of life, Give patients time to express, Listen carefully and give feedback, Verify patient information, Interpretation basis, Comfort and encourage patients, Adjust the way of interpretation, Identify patients’ efforts, Observe the patients’ suggestion, Maintain respectful tone, Make sure the patients have no problem, and Describe the treatment plan.

#### Preparation stage

The differences in courtesy with patients were statistically significant (90.17% vs. 97.81%; *P* = 0.002). Other communication before the performance showed no significant difference in the two assessment results.

#### Information collection

In the collection process, compared to the first assessment results, more interns were able to pay attention to the social, psychological or emotional factors associated with disease (37.16% vs. 10.98%; *P* < 0.001) and to discuss healthy lifestyle or disease prevention measures with patients (42.62% vs. 10.40%; *P* < 0.001). The results of the other projects were not statistically significant during the two-evaluation process.

#### Information given

Only a small number of interns were able to provide adequate patient information during doctor-patient communication. Of these, after completing the study, more interns were able to inform patients of their current physical condition (39.34%, vs. 8.67%, *P* < 0.001), including physical, laboratory, and diagnostic results. The other results were not statistically significant in the two-evaluation process.

#### Inquisition ending

There was no significant difference between the two assessments.

## Discussion

Network has brought dramatic changes to our life and added richness to learning styles [25–27]. Although the traditional teaching method in doctor-patient communication skills training was prevalent in medical schools and hospitals in China, its practicality has been challenged by the worsening doctor-patient relationship [7, 21, 22]. As the information technology advances, computer-aided learning has emerged and gained popularity as a complementary approach in education and training due to its convenience and interactivity [28–30]. Consequently, the study develops the doctor-patient communication learning software to create an effective and interactive learning environment for interns. In addition, the accompanying patient satisfaction questionnaire can combine course study with clinical practice. A comparison is made about the effect of our software on students’ learning and communication skill improvement between the pre-training and post-training sessions in the study.

According to the results, when obtaining information from patients, more interns allowed the patients sufficient time to describe their symptoms and share their perspectives. With the training and feedback from simulated patients (friends and family to play the role of patient), some students can recognize their deficiencies and pay more attention to patients gradually. Furthermore, the interns in this study also have more experiences in demonstrating medical care, concern, empathy and compassion, as well as the ability to communicate information effectively with patients. However, it is not indicated in the study that the interns significantly improved their ability to explain the disease more professionally because the relevant theoretical knowledge was not included in the software. Moreover, in terms of most of the learning and improvement of communication skills, the software does not show its superiority. It may be related to the monotonous settings of software problems, inadequate learning content and insufficient diversity of learning forms.

Medicine involves the integration of not only art and science but also magic and creative ability, and the building of a harmonious doctor-patient relationship reflects such artistic quality [8, 31, 32]. Communication and interpersonal skills for doctors, in particular young Chinese medical students, plays a big role. We hope that the application of this doctor-patient communication learning software turns out helpful and valuable.

The study was limited because of the use of fewer test questions and inadequate variety of the subjects. Some insufficiencies can also be found in the current study. Specifically, we plan to improve and refine our explorations along the following directions: First, establishment of an independent application; Second, addition and modification of questions; Third, addition of new learning patterns; Fourth, extending the user population (fresh doctors, nurses, and patients). Besides, in the preliminary application, only the self-controlled trial was made. In the subsequent study, a paired designed trial is expected to prepare to evaluate the software more fully based on current practices.

## Conclusions

We designed a type of learning software to train interns to advance their communication skills and evaluated its validity for improving doctor-patient communication in self-controlled trials. The self-controlled tests were implemented among 183 interns to assess the quality of communication skill acquisition by applying the new learning software. It is concluded that the doctor-patient communication software can provide a practice platform for interns. In the part of Patient Satisfaction Questionnaire, interns can acquire some feedback from patients, which allows them to know more about doctor-patient communication skills.

Project name: Teaching Reform Project of the First Affiliated Hospital of Xi’an Jiaotong University.

Project home page: http://yhgt.applinzi.com/

Operating system(s): Linux.

Programming language: PHP language.

Other requirements: Nginx 1.6.3 or higher, Thinkphp 3.2.3 or higher.

Any restrictions to use by non-academics: licence needed.

## Data Availability

The data is presented in the table and figures. Readers seeking access to any of the raw data, software code and test framework may contact the corresponding author, Qiling Li: liqiling@mail.xjtu.edu.cn License: This article is published under license to BioMed Central Ltd. This is an Open Access article distributed under the terms of the GNU General Public License (http://www.gnu.org/licenses/old-licenses/gpl-2.0.html), which permits unrestricted use, distribution, and reproduction in any medium, provided the original work is properly cited.

## Abbreviations

CST: Communication skills training
SEGUE: Set Elicit Give Understand End
SPs: Simulated patients
SPSS: Statistical Program for Social Sciences
